# A Fully Coupled Model for Electromechanics of the Heart

**DOI:** 10.1155/2012/927279

**Published:** 2012-10-16

**Authors:** Henian Xia, Kwai Wong, Xiaopeng Zhao

**Affiliations:** ^1^Department of Mechanical, Aerospace, and Biomedical Engineering, University of Tennessee, Knoxville, TN 37996, USA; ^2^Joint Institute for Computational Sciences, Oak Ridge National Laboratory, Oak Ridge, TN 37831, USA

## Abstract

We present a fully coupled electromechanical model of the heart. The model integrates cardiac electrophysiology and cardiac mechanics through excitation-induced contraction and deformation-induced current. Numerical schemes based on finite element were implemented in a supercomputer. Numerical examples were presented using a thin cardiac tissue and a dog ventricle with realistic geometry. Performance of the parallel simulation scheme was studied. The model provides a useful tool to understand cardiovascular dynamics.

## 1. Introduction 

Cardiovascular disease is the leading cause of death in America. Computer simulation and visualization of complicated dynamics of the heart have great potentials to provide quantitative guidance for diagnosis and treatment of heart problems. There have been intensive research efforts on developing accurate computer models to advance the understanding on the mechanisms of cardiovascular dynamics [1]. 

Inspired by the pioneering work of Hodgkin and Huxley [2], many mathematical models have been developed [3]. Meanwhile, a variety of mathematical models have been proposed for electromechanical simulations. Nash and Panfilov [4] presented a computational framework to couple a three-variable FitzHugh-Nagumo-type [5] excitation-tension model to governing equations of nonlinear stress equilibrium employing the electromechanical and mechanoelectric feedback. Niederer et al. [6] quantitatively characterized the binding of Ca^2+^ to TnC, the kinetics of tropomyosin, the availability of binding sites, and the kinetics of crossbridge binding after perturbations in sarcomere length. Gurev et al. [7] illustrated methods to construct finite element electromechanical models of heart and to develop anatomically accurate ventricular mesh based on magnetic resonance and diffusion tensor magnetic resonance imaging of the heart. The work of [7] focused on the construction of the ventricular meshes and did not consider the influence of the mechanical contraction on the cardiac electrophysiology. Göktepe and Kuhl [8] proposed an implicit and entirely finite element-based approach to the two-way coupled excitation-contraction problem. The electrophysiology was described by a FitzHugh-Nagumo-type (FHN) model in [8]. Doyle et al. [9] applied the parallel computing to the simulation of heart mechanics. They assessed the model's performance using an unstructured mesh, and they achieved the maximum speed-up factor as 15.9 when using 32 threads. Lafortune et al. [10] developed a parallel electromechanical model of the heart. Their model could run efficiently in hundreds of processors using a ventricular mesh of realistic geometry. Lafortune et al. described the electrophysiology by the simple three-variable FitzHugh-Nagumo-type (FHN) model [5] or the three-variable Fenton-Karma (FK) model [11] and employed a “one-way coupling” in which the displacements do not affect the electrophysiology. Moreover, the influence of the heart's mechanical behavior on its electrical behavior, which is termed as mechanoelectric feedback, has drawn researchers' attention [12–15]. Mechanoelectric feedback may be caused by stretch-activated channels or the influence of stretch on electrical signal propagation.

This work aims to develop a two-way coupled electromechanical model for parallel simulation of complex cardiovascular dynamics. Towards that aim, we have developed a fully coupled electromechanical model of the heart, which integrates the cardiac electrophysiology, the cardiac mechanics and the two-way coupling arising from the excitation-induced contraction and the deformation-induced generation of current. The cardiac electrophysiology is described by the Beeler-Reuter (BR) model [16]. The coupled electrical and mechanical problem is solved implicitly using finite element method. The computational algorithm is parallelized using the message passing interface (MPI). The model is tested by simulating a thin cardiac tissue and a dog ventricle with realistic geometry. 

This paper is organized as follows. In Section 2, we introduce the physiological models. The numerical computation approach to the coupled problems is introduced in Section 3.  Section 4 shows the numerical results. We discuss the results and conclude the paper in Section 5.

## 2. Physiological Models

**Figure 1:**
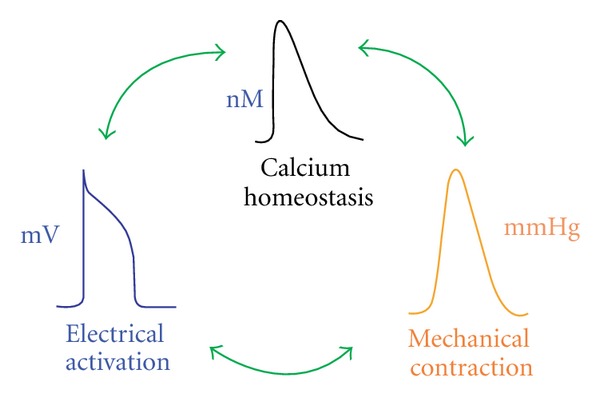
Schematic representation of the coupling between electrical, chemical, and mechanical functions of the heart.

Hearts beats are the result of a sequence of electrochemical excitation waves that are initiated from the sinoatrial node. The electrical impulses induce intracellular calcium cycling, which in turn causes heart muscle to contract. This process, known as excitation-contraction coupling (ECC), is essential to understanding of the heart. On the other hand, mechanical changes that response to neural and hormonal influences also impact on the electrical properties. This complementary concept is called mechanoelectric feedback. See Figure 1 for the relation between electrical activation, chemical homeostasis, and mechanical contractions.

### 2.1. Cardiac Electrophysiology

Dozens of models have been proposed over years to simulate cardiac electrophysiology [3]. Most of those models are drawn from the pioneering work of Hodgkin and Huxley [2]. In this work, the Beeler-Reuter (BR) model [16] is adopted for numerical illustrations. The BR model describes the transmembrane voltage in a single cell as follows

(1) $$\begin{array}{c} \frac{dv}{\partial t}=-\frac{I_{\text{ion}}}{C_{m}}, \end{array}$$

where, *v* represents transmembrane voltage, *C*_*m*_ represents membrane capacity, and the total current is described as:

(2) $$\begin{array}{c} I_{\text{ion}}=I_{\text{Na}}+I_{K1}+I_{x1}+I_{\text{Ca}}+I_{\text{sac}}-I_{\text{stim}}. \end{array}$$

Here, *I*_Na_ represents the voltage-gated Na current, *I*_*K*1_ represents the time-independent outward current, *I*_*x*1_ represents the time-activated outward current, *I*_Ca_ represents a slow inward current, and *I*_stim_ represents the external stimulation. Note that the original BR model does not include *I*_sac_, the stretch-activated channel, whose details will be discussed later. The stimulus current *I*_stim_ is selected to be a square wave pulse of −80 *μ*A/*μ*F for 1 ms. We refer readers to [16] for details of the BR model. 

In cardiac tissue, (1) is extended into a reaction-diffusion form to include spatial diffusion of currents:

(3) $$\begin{array}{c} \frac{\partial v}{\partial t}+\frac{I_{\text{ion}}}{C_{m}}-\nabla_{x}\cdot \left(D\cdot \nabla_{x}v\right)=0, \end{array}$$

where **x** represents the spatial coordinate of each material point in the heart; **D** is the diffusion tensor, which controls the transduction orientation and speed of the electrical wave of excitation in the cardiac tissue; *C*_*m*_ is the membrane capacitance and is set as 1 *μ*F/cm.

### 2.2. Cardiac Mechanics

We denote the initial configuration (diastole) of the heart by Ω_0_  and the deformed configuration (systole) by Ω. The position vector of a material point in the initial configuration is given by  **X** = **X**_*i*_ **e**_*i*_, where  **e**_*i*_  are the unit base vectors of a rectangular Cartesian coordinate system. Denote the position of the material point  **X**  at time *t* by  **x** = **x**_*i*_ **e**_*i*_. Then, the spatial coordinate of a material point  **X** can be represented by **x** = Φ(**X**, *t*). The function Φ can be regarded as a map between the initial configuration and the configuration at time *t*. The two measures, **x**  and  **X**, are related by the deformation gradient, as shown in (4). Consider the following:

(4) $$\begin{array}{c} F=\frac{\partial x}{\partial X}. \end{array}$$

There are two approaches in describing the deformation of a continuum: the Lagrangian description uses the material coordinates **X** as independent variables and the Eulerian description uses the spatial coordinates **x**  as independent variables [17]. The Eulerian description is often adopted for fluid dynamics. In this work, the Lagrangian description approach is utilized. There are two formulations for the Lagrangian approach: total Lagrangian formulation and updated Lagrangian formulation. In the total Lagrangian formulation, equations are discretized with respect to the original configuration. In contrast, the updated Lagrangian formulations are based on the current configuration and are commonly used for nonlinear, large deformations. Thus, we use the updated Lagrangian formulation in this work. 

For each time step Δ*t*, the displacement of a material point, denoted by **u**(**X**, *t*), is defined by the difference between its current position and its previous position (5). The displacement **u**(**X**, *t*) is governed by the equilibrium of the linear momentum, and the equation is described as (6). In (6), **σ** is the Kirchhoff stress tensor; **b** accounts for the body force or externally applied stresses. The Kirchhoff stress  **σ**  is composed of a passive component **σ**_**pass**_ and an active component **σ**_**act**_. The active force **σ**_**act**_ is generated from the electrical excitation and will be explained later in detail. The passive component **σ**_**pass**_ is determined by the equation of the elementary mechanics (7). In (7), **χ** = 0.5 MPa  and *ζ* = 0.2 MPa are the Lame constants which govern the isotropic stress response; *ξ* = 0.1 MPa represents the passive stiffness of myofibers. The left Cauchy-Green tensor is denoted as **p** and is defined as (8). The parameter values were referred to the work of Nash and Panfilov (2004) [4] and Göktepe and Kuhl (2010) [8]. Consider

(5) $$\begin{array}{c} u\left(X,t\right)=\Phi \left(X,t\right)-\Phi \left(X,t-\Delta t\right), \end{array}$$

(6) $$\begin{array}{c} \nabla_{x}\cdot \sigma +b=0, \end{array}$$

(7) $$\begin{array}{c} \sigma_{pass}=\left(\frac{\chi }{2}\ln A-\zeta \right)+\zeta p+2\delta \xi \left(B-1\right)\kappa , \end{array}$$

(8) $$\begin{array}{c} p=FF^{T}. \end{array}$$

Let us denote the local orientation of a myofiber at initial configuration by a unit vector **a**_0_ and that at deformed configuration by vector **a**. In (7), **κ** represents the deformed structural tensor and is defined as (9). In (9), **κ**_0_ is the structural tensor at initial configuration. The structural tensors **κ**_0_ at initial configuration and **κ** at deformed configuration represent dominating directions in a specified neighborhood of a node [18, 19]. Consider the following:

(9) $$\begin{array}{c} \kappa =a⊗a=F\kappa_{0}F^{T}. \end{array}$$

In (7), the symbol *δ* denotes the coefficient that determines whether or not the stiffness of the myofibers is in effect. It indicates that when there is stretch at a material point, *δ* will be 1, otherwise it is 0. Mathematically, it is defined as (10). In (10), |**a**| represents the stretch at a material point of the heart. At initial configuration, |**a**_0_| = 1, while at deformed configuration, there may be stretch at some material points that causes |**a**| > 1. Moreover, the scalars *A* and *B* in (7) are defined as ([Disp-formula EEq11]). Consider

(10) δ={1, if  |a|>10, otherwise,

(11) A=det⁡⁡(FTF)

### 2.3. Electromechanical Coupling

Summarizing Sections 2.1 and 2.2, the coupled problems are governed by the following equations:

(12) ∂v∂t−∇x·(D·∇xv)+IionCm=0,∇x·σ+b=0,

(13) ∂v∂n=∇x·n=0,σ·n=0,x=x¯.

Equations (12) show the full coupling of the cardiac electrophysiology and the cardiac mechanics. The membrane potential and the spatial coordinates of each node are solved simultaneously from (12). The first equation of (13) represents the no-flux boundary condition imposed on the surface domain of the heart denoted by ∂Ω. The symbol **n** is the outward surface normal on ∂Ω. The second equation of (13) defines the natural boundary condition imposed on ∂Ω. The third equation of (13) shows the essential boundary condition imposed at points which are fixed to ensure that the mechanical problem is well defined. The domain where the essential boundary condition is imposed on is denoted by ∂Λ.

#### 2.3.1. Excitation-Induced Contraction

In Section 2.2, we introduced the active Kirchhoff stress **σ**_**act**_ · **σ**_**act**_ is generated by the excitation-induced contraction. From the perspective of geometry, the direction of the active Kirchhoff stress should be determined by the structural tensor **κ**, and its magnitude is controlled by the transmembrane potential *v*. Let the magnitude of the active stress be *f*(*v*), we have **σ**_**act**_ = *f*(*v*)**κ**. 

Quite a few models have been proposed to simulate the voltage-dependent active fiber tension*f*(*v*) [4, 18]. In this work, we adopt the simplified equation proposed by [4]:

(14) f˙=ε(v)[kf(v−vr)−f]ε(v)=ε0+(ε∞−ε0)exp⁡⁡[−exp⁡⁡(−l(v−v¯))].

**Figure 2:**
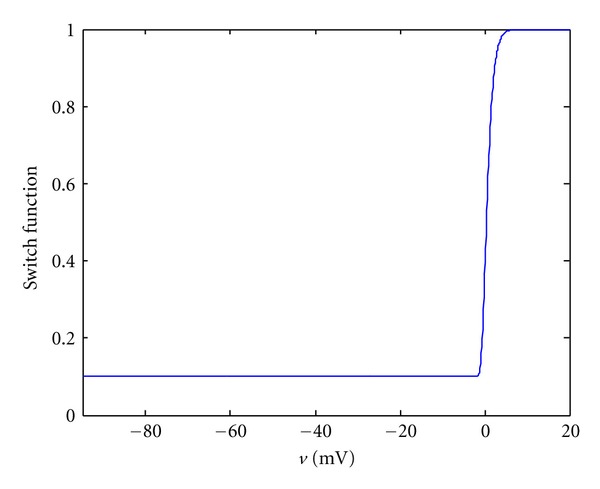
Illustration of the switch function with the following parameters: *ε*_0_ = 0.1/mV, *ε*_*∞*_ = 1/mV, l = 1/mV, $\bar{v}=0\;\text{mV}$.

In (14), the symbol *k*_*f*_ = 0.005 MPa/mV is the maximum active fiber tension, and *v*_*r*_ is the resting potential which is about −94.7 mV for cardiac cells in the BR ionic model. The switch function is denoted by *ε*(*v*) and it determines how fast the active fiber tension will change with respect to the transmembrane potential *v*. Parameters' values are *ε*_0_ = 0.1/mV, *ε*_*∞*_ = 1/mV, l = 1/mV, $\bar{v}=0\;\text{mV}$. When *v* changes from −94.7 mV to 20 mV, the function is as Figure 2.

Note that the calcium fluctuation is not studied in this work and the active fiber tension is controlled by the membrane potential directly for simplification, as shown in (14).

#### 2.3.2. Diffusion Tensor

In (3), the interconnection between cells is regulated by the diffusion tensor **D**. It controls the transduction speed of the electrical wave of excitation in the cardiac tissue. Due to the anisotropic properties of the heart tissue, it is observed in experiments that the conduction is obviously faster in the myofiber directions than in other directions. To consider the additional speed along the fiber orientations, the diffusion tensor is split into two parts: **D** = *d*_iso_ **I** + *d*_ani_ **κ**. The symbol **I** denotes an identity matrix. The coefficient *d*_iso_ = 0.001 cm^2^/ms controls the speed of the isotropic transduction to all directions and the coefficient *d*_ani_ = 0.0001 cm^2^/ms denotes the additional speed along the fiber orientations. Since the structural tensor **κ** is dependent on the spatial coordinate, the diffusion tensor at each material point will change with the reshaping of the heart.

#### 2.3.3. Deformation-Induced Generation of Current

In (3), the total ionic transmembrane current *I*_ion_ consists of a component *I*_sac_. The stretch activated channels are the ion channels which open their pores in response to mechanical deformation of the cell membrane [19]. According to what mechanisms the current is induced, there are different kinds of formulations for the stretch activated channels. In this work, we employ the formulation proposed by [20]:

(15) $$\begin{array}{c} I_{\text{sac}}=\delta G_{s}\left(\left|a\right|-1\right)\left(v-v_{s}\right). \end{array}$$

In (15), *G*_*s*_ = 10 mS/*μ*F is the maximum conductance; *v*_*s*_ = −20 mV is the resting potential of the stretch-activated channels; *δ* is the coefficient that determines whether or not the stiffness of the myofibers is in effect, as defined in (10); |**a**| is the stretch at a material point of the heart. 

## 3. Numerical Computation Approach

The governing equations (12) are solved using the operator splitting method [21]. First, we solve the following nonlinear ordinary differential equation using the forward Euler method [22]:

(16) $$\begin{array}{c} \frac{dv}{dt}+\frac{I_{\text{ion}}}{C_{m}}=0. \end{array}$$

Then, the solution from (16) is used to solve the following partial differential equation in (17) using the implicit Euler method. These two equations are solved iteratively for each time step. Consider the following:

(17) ∂v∂t−∇x·(D·∇xv)=0∇x·σ+b=0.

Weak forms of (17) are constructed following the classical Galerkin procedure. The weak form is obtained by taking the product of (17) with the test functions *δ* **x** and *δv* and integrating them over the domain. The time independent test functions are required to be C^0^ and satisfy the essential boundary conditions on ∂Ω. Multiplying the test function *δ* **x** and *δv* with the two equations in ([Disp-formula EEq18]) and carrying out integration by part yield

(18) Gx=∫Ω∇x(δx):σdV−∫∂Ωδx·σ·nda−∫Ωδx·bdV=0,Gv=∫Ω[δv∂v∂t+∇x(δv)·(D·∇xv)]dV−∫∂ΩδvD·∇xv·nda=0.

Applying the natural boundary conditions to ([Disp-formula EEq18]) leads to

(19) Gx=∫Ω∇x(δx):σdV−∫Ωδx·bdV=0,Gv=∫Ω[δv∂v∂t+∇x(δv)·(D·∇xv)]dV=0.

At each time step, ([Disp-formula EEq19]) are linearized as follows:

(20) Gx(xn+1,vn+1)=Gx(xn,vn)+ΔGx(xn,vn;xn+1−xn,vn+1−vn),Gv(xn+1,vn+1)=Gv(xn,vn)+ΔGv(xn,vn;xn+1−xn,vn+1−vn).

We can then solve for Δ**x** = **x**_*n*+1_ − **x**_*n*_ and Δ*v* = *v*_*n*+1_ − *v*_*n*_ from the linearized equations. 

The conventional isoparametric Galerkin procedure is followed to discretize the continuous weak form equations. The domain of the heart *Ω* is decomposed into subdomains *Ω*_*e*_^*h*^, and each subdomain is an element. Then the field variables **x** and *v*, and the two associated test functions are interpolated in each subdomain as

(21) xeh(X,t)=∑j=1nenNj(X)xje(t),veh(X,t)=∑j=1nenNj(X)vje(t)

In ([Disp-formula EEq21]), *n*_en_ is the number of nodes per element and *N* ^*j*^(**X**) is the C^0^ interpolants, often called shape functions in finite element literatures. The implicit Euler method is utilized when discretizing time derivative terms in ([Disp-formula EEq18]). Finally, we achieved a linear system equation in form of:

(22) {A}4N∗4N(ΔxnΔynΔznΔvn)n=1~N={b}4N.

The linear system equation has a degree of 4∗*N*, where *N* is the number of the nodes in the mesh. For each node, (Δ*x*, Δ*y*, Δ*z*, Δ*v*) are solved. They are then used to update the membrane potential and the spatial coordinate of each node. 

The model was implemented in C++ and was parallelized using the message passing interface (MPI) [23]. An open source software package called METIS [24] was used to partition the heart mesh so that computational loads are balanced among CPUs. The algorithms in METIS were based on multilevel recursive-bisection, multilevel k-way, and multiconstraint partitioning schemes. 

Two parallel solvers were used to solve the final linear system. The two solvers are the hierarchical iterative parallel solver (HIPS) [25] and the solver from the Trilinos package [26]. Both of them implemented the generalized minimal residual method [27]. When using the Trilinos, the linear system was preconditioned by the Jacobi preconditioner. Simulations were run on the supercomputer, Kraken [28]. The open source software VisIt [29] was used for visualization.

## 4. Numerical Results

### 4.1. A Thin Cardiac Tissue

We first conducted simulations in a thin square cardiac tissue of the size 0.4 ∗ 0.4 ∗ 0.001 cm^3^. In simulations, the top-left and the bottom-right corners were fixed, and the fiber orientation was along vertical direction. We used the Forward Euler method to solve the ODE in (16) at a time step of 0.005 ms. The PDEs in (17) are solved using the implicit Euler method with a time step of 0.1 ms. 

**Figure 3:**
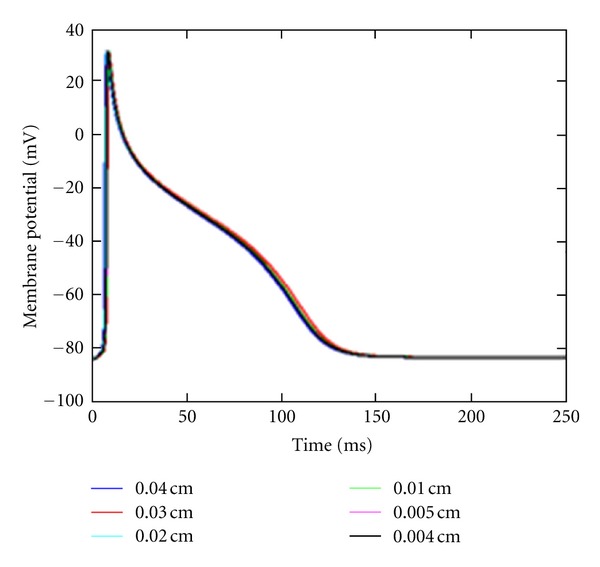
Action potential of a node at (−0.2 cm, −0.2 cm, 0.0005 cm). Mesh size was decreased from 0.04 cm (blue) to 0.004 cm (black).

Action potential of a node at (−0.2 cm, −0.2 cm, 0.0005 cm) as shown in Figure 3 was obtained using different mesh sizes. The numerical results show that consistent action potential responses are obtained using different mesh sizes. Because the cardiac tissue is very thin, it is treated like a 2d tissue. The mesh size is with respect to the *x* and *y* directions which are each 0.4 cm in length.

**Figure 4:**
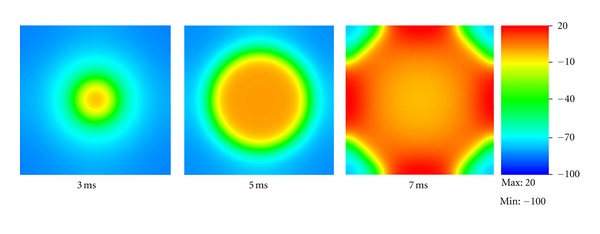
Electrical wave propagation in a piece of heart tissue.

We then performed an electromechanical simulation in the tissue (0.4 cm ∗ 0.4 cm ∗ 0.001 cm) with mesh size = 0.004 cm. The stimulation was imposed at the center of the tissue.  Figure 4 shows the electrical wave propagation without considering the contraction. The electrical wave propagated symmetrically from the center to the whole tissue. This test validates the part of “reaction-diffusion” in our model by reproducing the basic phenomenon of electrical wave propagation in a square tissue. Note that the coefficient *d*_ani_ was set to 0 in this simulation so that the tissue was isotropic.

**Figure 5:**
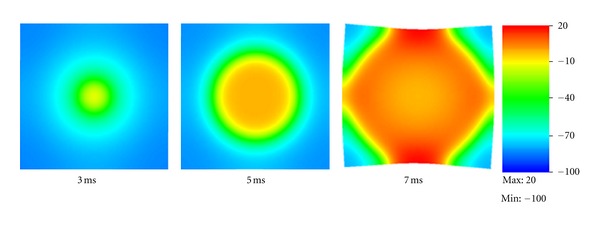
Electrical wave propagation and excitation-induced contraction in a piece of heart tissue. Although these tests are simple and straightforward, they demonstrate the model's capability of performing fully-coupled electromechanical simulations.

Figure 5 shows the electrical propagation as well as the excitation-induced contraction in a tissue. In this test, the fibers of the tissue were aligned vertically. Thus, the tissue deformed in vertical direction, as clearly shown at time *t* = 7 ms in Figure 5. Moreover, in contrast to the previous test, we considered the additional speed along the fiber orientations, as indicated by  **D** = *d*_iso_ **I** + *d*_ani_ **κ**. The coefficient *d*_ani_ was set to 0.0001 cm^2^/ms. It is easily observed from Figure 5 that the propagation was obviously faster in vertical direction. 

### 4.2. Dog Ventricle with Realistic Geometry

**Figure 6:**
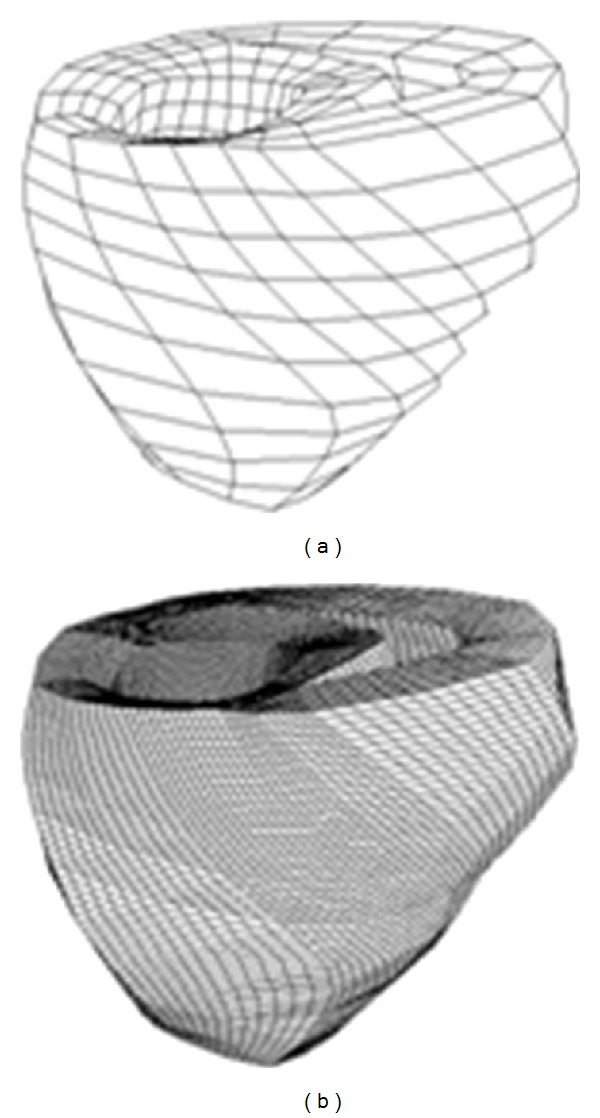
Dog ventricle mesh: original (left) and refined (right).

We also simulated the contraction of a dog ventricle with realistic geometry. Two meshes were examined. The first mesh consisted of 880 Hexahedron elements. The second mesh which had 190080 hexahedron elements was refined from the first one using the software CUBIT which was developed at Sandia National Laboratories [30]. See Figure 6 for the original mesh and the refined one. The ventricle mesh was obtained from [31].

**Figure 7:**
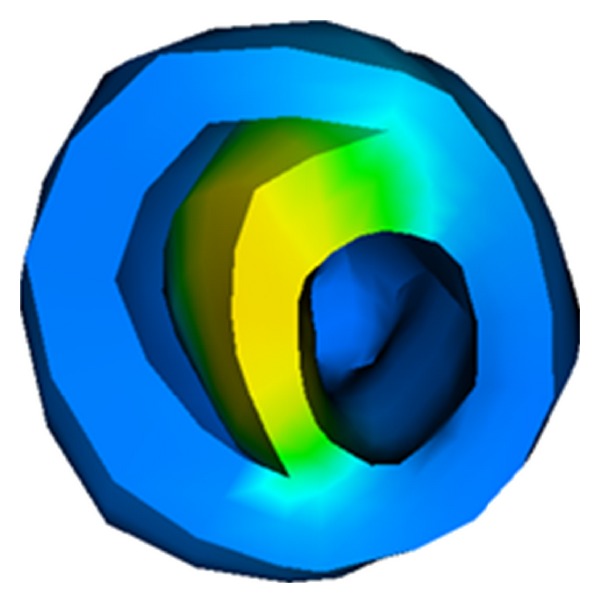
Initial stimulus: the stimulus was imposed on the superior section of the ventricle.

The constitutive and coupling models, the anisotropic electrical conductivity, and other parameters were the same as used earlier. The stimulus was imposed on the superior section of the ventricle as shown in Figure 7. Some nodes on the upper surface were restrained so that the problem would be well defined. We assumed that the normal of the fiber at any point is pointing to the geometric center. Under this assumption, the fibers form layers of muscle in the heart. We note that this assumption may not be close to realistic fiber layouts in the heart. However, the assumed structure allows us to test the efficiency and robustness of the computational algorithms on a heart, on which fiber orientation changes from point to point. 

**Figure 8:**
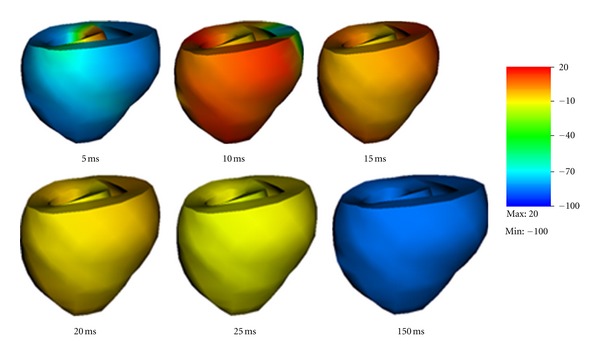
Electromechanical simulation of a dog ventricle with realistic geometry.

Figure 8 shows an electromechanical simulation of a dog ventricle with realistic geometry. Electrical stimulation was imposed on the upper surface of the septum. The contraction state kept for about 100 ms and then slowly recovered to the resting state.

**Figure 9:**
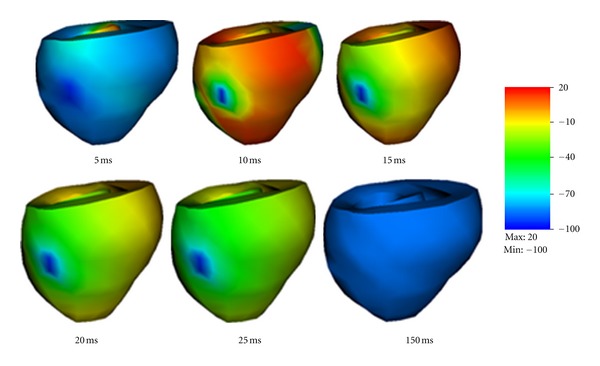
Electromechanical simulation of a dog ventricle with a scar near the outer surface.

We also simulated a ventricle with a scar near the outer surface. We have adopted a simplified description of scar. Specifically, we assumed the scar has no conduction capability and can maintain passive mechanical contractions like other cells. The scar had a size as shown in Figure 9. The scar has a radius of about 1cm in the surface area and its thickness was similar to the ventricle wall. 

Figure 9 shows the shapes of the contracting ventricle and the spatial distribution of membrane potentials at six moments. Comparing Figure 9 with Figure 8, obvious difference can be observed. When the scar was present the membrane potentials was evidently smaller, and this could cause smaller active fiber tension and thus weakened pumping ability. Although no serious physiological conclusion could be given in this study since our simulations ware preliminary and lacked experimental validation, the simulations demonstrated the capability of our model to study the real heart.

### 4.3. Performance Analysis

**Figure 10:**
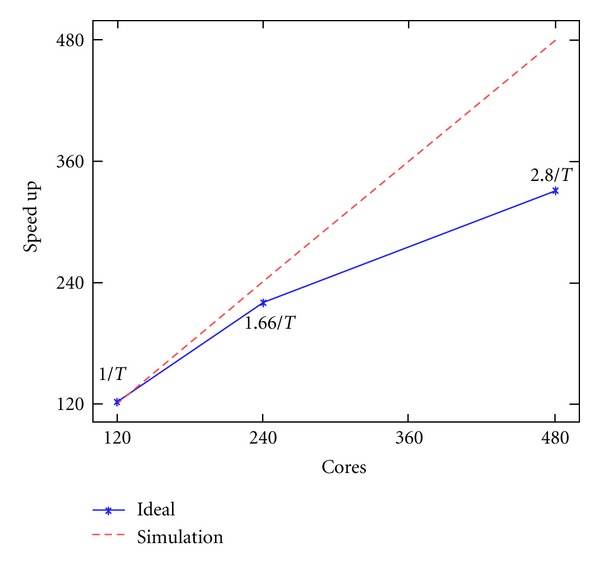
Scalability up to 480 CPUs on the Kraken system.

Parallel efficiency is crucial for our model. The requirements of using meshes with hundreds of thousands of elements to achieve high accuracy and resolution make the computational efficiency a great challenge. The parallel efficiency is measured by the analysis of the scalability. A mesh with 1.56 million Hexahedron elements was used. In this analysis, we used 120, 240, and 480 cores. The time spent when using 120 CPUs was taken as the reference value.  Figure 10 shows the strong scalability up to 480 cores on the Kraken supercomputer. In this case, the scalability for 240 cores was about 90% of the ideal and the scalability for 480 cores was about 70% of the ideal. The decrease of the scalability was due to the increase of the proportion of amount of communications per iteration and the increase of the proportion of the number of ghost nodes. The scaling ability was limited by the performance of the PDE solver from the Trilinos package [26]. In future studies, the performance will benefit from choosing a better parallel solving algorithm. Also the performance may be improved if we take use of the CUDA [32] which is a parallel computing platform and programming model invented by NVIDIA. 

## 5. Discussions and Conclusion

It is a common approach in the literature to solve the electromechanics problem in an iterative manner. In each step, the electrical problem is solved first and then the results from the electrical solution are submitted into the mechanical problem, whose solution is then used to solve the electrical problem in the next step. Since the electrical problem and the mechanical problem are solved separately, it is also a common practice to adopt a fine mesh for the electrical field and a much coarser mesh for the mechanical field. 

The contribution of this work is to develop a fully coupled scheme to accurately solve the electromechanics of the heart. We have developed a cardiac electromechanics model, which integrates cardiac electrophysiology, mechanical contraction, as well as their interactions. Realistic physiological models have been adopted to describe the electrical and mechanical functions in the heart. The model has been numerically solved using an implicit, finite element-based approach. Numerical simulations have been conducted using parallel simulation in tissues of different geometries. The cardiac mechanics is described by the updated Lagrangian approach, which views the problem from the current configuration and takes derivatives and integrals with respect to the spatial coordinates. In perspective of mesh description, the updated Lagrangian description is characterized by making the material points remain coincident with mesh points. Therefore, the Lagrangian description simplifies the imposition of boundary conditions since the element boundaries of the mesh remain coincident with material boundaries. The developed model and computer codes have been validated at each step using simple test examples to ensure accuracy in numerical computations. Multiple simulations have been conducted using various meshes and parameters to ensure numerical robustness of the developed model. 

Since the computations involve millions of nodes, the current framework has limitations for real-time applications. In future studies, we will further improve the performance of the model using more efficient PDE solvers or implementing the model in CUDA [32]. This paper has adopted a simplified fiber configuration, where the normal of the fiber was assumed to point toward the geometric center of the heart. More realistic heart shape and fiber configurations should be utilized in future work for physiologically faithful parameter studies. Moreover, since Lagrangian meshes deform with material, the mesh may become distorted if the deformation of the heart is too large.

Future work may also consider more detailed models for active stresses such as the hybrid model [18]. In the hybrid model, the active force is dependent on the [Ca^2^+]_i_, rather than the transmembrane potential, and this is more biophysically reasonable. Moreover, the hybrid model also takes into consideration binding of intracellular Ca^2+^ to troponin C, configuration change of tropomyosin, and interaction of actin and myosin, which is a more accurate description on excitation contraction interaction. Numerical simulations have been carried out for purpose of validating the model implementation in this paper, but more numerical experiments will be executed using the platform to investigate the interaction of electrical and mechanical functions in the heart and their influences to cardiac arrhythmias.
